# Determinants of Adolescent Sexual and Reproductive Health in Sub-Saharan Africa: Protocol for an Umbrella Review

**DOI:** 10.2196/51278

**Published:** 2023-11-17

**Authors:** Mona Ziba Ghadirian, Khalid Omer, Anne Cockcroft

**Affiliations:** 1 Department of Family Medicine McGill University Montreal, QC Canada

**Keywords:** adolescents, youth, sexual health, reproductive health, sexually transmitted infections, pregnancy, sub-Saharan Africa

## Abstract

**Background:**

Adolescents are a large proportion of the population in sub-Saharan Africa and face multiple risks to their health and well-being*.* Prior systematic reviews have focused on specific adolescent sexual and reproductive health outcomes such as teen pregnancies, HIV/AIDS, and sexually transmitted diseases. A comprehensive synthesis of the influential factors that shape different aspects of adolescent sexual and reproductive health can inform health policy and program development for this important segment of the population.

**Objective:**

This paper presents the protocol for an umbrella review that aims to synthesize the existing knowledge in the literature on the associations among individual, family, and societal factors and sexual and reproductive health outcomes among adolescents in sub-Saharan Africa.

**Methods:**

We will include systematic reviews that identify factors associated with sexual and reproductive health outcomes among adolescents, 10-19 years of age, in sub-Saharan Africa. Reviews can include quantitative and qualitative primary studies with or without meta-analysis. Academic and gray literature searches will identify reviews from PubMed, Scopus, CINAHL, Cochrane Database of Systematic Reviews, ProQuest, Google, and Google Scholar. Two reviewers (MZG and KO) will independently carry out title, abstract, and full text screening, assess methodological quality, and extract data. We will assess the methodological quality of the included studies using the Joanna Briggs Institute standard forms. The review will present findings in narrative form and in tables and will follow PRISMA (the Preferred Reporting Items for Systematic Reviews and Meta-Analyses) reporting guidelines.

**Results:**

A preliminary search in April 2023 found 1351 articles to be screened.

**Conclusions:**

This umbrella review will permit a comprehensive and high-level understanding of the various factors that influence adolescent sexual and reproductive health in sub-Saharan Africa.

**Trial Registration:**

PROSPERO International Prospective Register of Systematic Reviews CRD42023394512; https://www.crd.york.ac.uk/prospero/display_record.php?RecordID=394512

**International Registered Report Identifier (IRRID):**

DERR1-10.2196/51278

## Introduction

Adolescence is the transition phase between childhood and adulthood that serves as a window of opportunity for development and growth. Different exposures help adolescents establish patterns of behaviors related to diet, physical activity, substance use, and sexual activity. These behaviors and influences, especially related to adolescent sexual and reproductive health (ASRH), can either protect or put at risk their health and the health of others around them now and in the future [1]. During the past 25 years, following the International Conference on Population and Development in Cairo (Egypt), there has been improvement in ASRH outcomes globally [2]. However, progress is unequal between and within countries and in different regions, such as Sub-Saharan Africa [3]. Adolescent girls in sub-Saharan Africa face an elevated risk of early marriage [4], teenage pregnancies [5], and exposure to unsafe abortions [6], leading to a high rate of maternal deaths among this group [7]. Lack of knowledge about safe sexual and reproductive behaviors and practices [8,9] exposes adolescents to HIV and sexually transmitted infections [10]. The risks are higher among rural girls who are poor and uneducated [4]. Multifaceted barriers at the individual, family, community, service, and policy levels still impede ASRH policies and programs in the provision of comprehensive information and services to adolescents [11-13]. Attitudes of peers, parents, family members, and service providers influence adolescents’ access to ASRH information and services [14]. In many settings, discussion about ASRH and the use of related services, especially by unmarried adolescents, is stigmatized.

To improve ASRH and address inequalities, policy makers and planners need to set priorities based on robust evidence. Historically, statistics about adolescents have often been merged with children or adults even in national databases [15,16]. There has been a steady growth in published evidence, including systematic reviews, about ASRH in sub-Saharan Africa. However, studies and reviews focus on specific ASRH outcomes (such as the use of contraceptives [17], teen pregnancies [18,19], transactional sex [20], HIV/AIDS [21], and other sexually transmitted diseases [STDs]) or interventions [22]. There remains a need for a comprehensive and overarching review of factors associated with the range of ASRH outcomes. In our search of the 3 main systematic review registries (the PROSPERO [International Prospective Register of Systematic Review], Joanna Briggs Institute Systematic Review Register, and Open Science Framework Registries), we did not find any umbrella review registered on this topic.

This umbrella review will identify and summarize evidence on the factors associated with the range of ASRH outcomes in sub-Saharan Africa. The aim is to identify and synthesize existing knowledge on the associations between individual, family, and societal factors and ASRH outcomes. The research questions are (1) what are the overlapping and unique factors associated with a broad range of ASRH outcomes (including teen pregnancies, transactional sex, sexual abuse, gender violence, HIV/AIDS, and other STDs)? (2) What are the research gaps in the literature around ASRH in sub-Saharan Africa? The intention is to cover a wide range of ASRH outcomes, using previously synthesized evidence in systematic reviews. The review will form part of the evidence considered in a larger study to co-design strategies to promote and protect ASRH in Bauchi State, Nigeria [23].

## Methods

### Study Design

The umbrella review [24] will examine the documented associations between ASRH outcomes including teen pregnancy, transactional sex, sexual abuse, gender violence, HIV/AIDS, and various STDs, and individual, family, and societal factors. There are many preexisting reviews of factors related to specific ASRH outcomes; we will not conduct a review of primary studies. The unit of search and data analysis will be systematic and scoping reviews, with or without meta-analyses [25]. By using an umbrella review approach, we can cover a wide range of ASRH outcomes. An umbrella review will permit a more comprehensive and higher-level analysis of the various factors that influence ASRH in sub-Saharan Africa. We seek to synthesize the available evidence about factors associated with each ASRH outcome. We will examine whether the results and conclusions of individual reviews of factors related to specific ASRH outcomes are consistent or contradictory. This review will provide an overarching view of current knowledge about the multifaceted issue of ASRH and will identify research gaps in the literature.

### Search Strategy

A comprehensive search strategy included both peer-reviewed articles and gray literature. We searched for peer-reviewed articles in PubMed, Scopus, CINAHL, and Cochrane Database of Systematic Reviews on April 11, 2023. The search matrix included a combination of controlled vocabulary and keywords to represent reviews, adolescents, sexual and reproductive health outcomes, and sub-Saharan Africa. We used a similar search matrix in the gray literature database ProQuest to search for relevant theses or dissertations. The gray literature search also used the Google and Google Scholar search engines with the search terms such as adolescent sexual reproductive Africa review. We included the first 10 search results from Google and Google Scholar in the initial screening. The initial searches considered published articles and gray literature irrespective of date of publication.

### Eligibility Criteria

The review will include systematic and scoping reviews of both quantitative and qualitative primary studies, with or without meta-analyses. All included reviews must describe a reproducible and systematic search strategy, well-defined inclusion and exclusion criteria and outcome, and risk of bias assessment of included primary articles. We will include academic and gray literature in English or French, irrespective of the date of publication. The population of interest is adolescents, 10-19 years of age, in sub-Saharan Africa. This includes studies irrespective of gender, place of residence (urban and rural demographics), and educational attainment (students and out-of-school youth). We will include reviews that include children, adults, or countries outside of sub-Saharan Africa if they present disaggregated data on youth (10-19 years of age) and sub-Saharan Africa. We will exclude studies of specific subpopulations, such as athletes or orphans. Textbox 1 describes the inclusion and exclusion criteria for this umbrella review.

**Inclusion and exclusion criteria for an umbrella review of factors related to adolescent sexual and reproductive health in sub-Saharan Africa.**

**Inclusion criteria**

- Reviews published in peer-reviewed journals, thesis manuscripts, and reports
- All reviews irrespective of date of publication and date range of search
- Articles written in English or French
- Quantitative and qualitative reviews. Scoping and systematic reviews, with or without meta-analyses. Reviews must include a reproducible and systematic search strategy, well-defined inclusion and exclusion criteria and outcome, and risk of bias assessment of included primary articles
- The review should include male and female adolescents, 10-19 years of age, or present disaggregated data for adolescents
- The review should include studies from sub-Saharan Africa or present disaggregated data from a country or countries in sub-Saharan Africa
- The domain under investigation is adolescent sexual and reproductive health. Adolescent sexual and reproductive health outcomes include issues around teen pregnancy, transactional sex, gender violence, sexual abuse, and the transmission of HIV and other sexually transmitted diseases. This review will consider any outcome that the authors explicitly state is related to adolescent sexual and reproductive health

**Exclusion criteria**

- Blogs, websites, and conference papers
- Reviews without a systematic and reproducible methodology for article identification, such as well-defined eligibility criteria and outcome of interest
- Reviews that do not provide the list of included primary studies
- Reviews concerned with specific populations such as adolescent athletes or patients

### Outcome

The primary domain of interest is outcomes related to ASRH. ASRH refers to more than the absence of disease. It also includes the physical, mental, and social well-being of adolescents such as the ability to access sexual health services, the prevention of unintended pregnancies, safe abortion, the prevention of STDs including HIV/AIDS, and all forms of sexual violence and coercion. This umbrella review will consider any outcome that has been explicitly defined by the authors as being related to ASRH. All included reviews will describe the individual, family, or societal factors associated with an ASRH outcome. Reviews may include studies of interventions to address ASRH outcomes such as school-based sex education initiatives, behavior change interventions, programs, and policies.

### Study Selection

We will combine reviews identified in the academic and gray literature searches and remove duplicates. Two reviewers (MZG and KO) will independently screen all articles. In the first round of study selection, 2 reviewers (MZG and KO) will independently read titles and abstracts and select potentially eligible articles. They will discuss and resolve disagreements on selection, with articles retained if there is no agreement.

**Figure 1:**
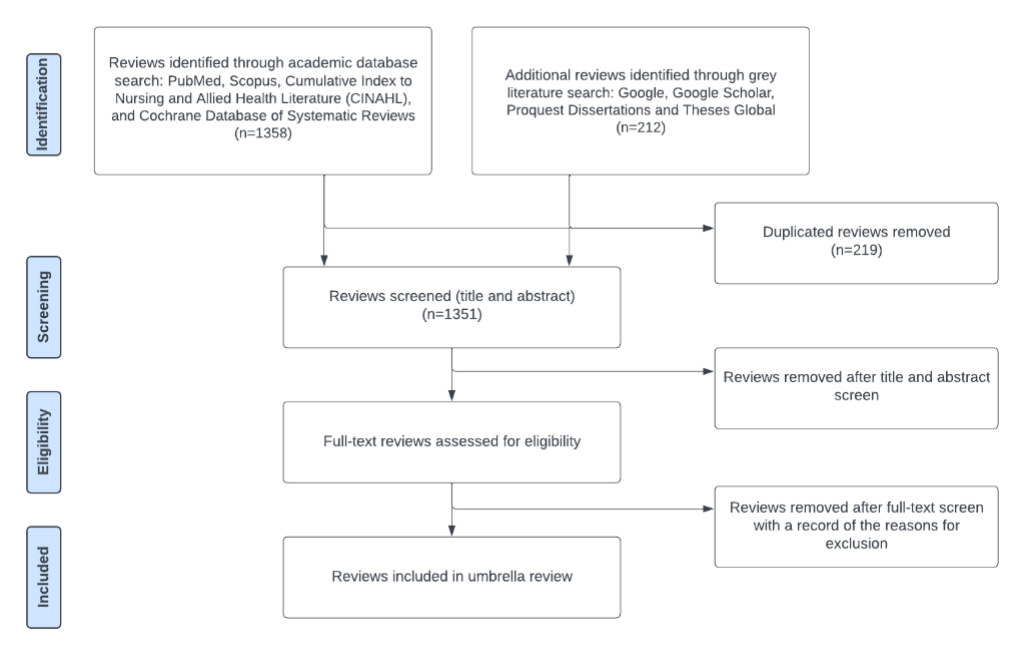
Flow diagram illustrating the study selection process of review articles in an umbrella review of the determinants of adolescent sexual and reproductive health in sub-Saharan Africa.

In the second round of study selection, 2 reviewers (MZG and KO) will screen full-text articles of the remaining reviews from the first round, according to the eligibility criteria described above. The 2 reviewers will agree on whether to include or exclude each article and on the primary reason for exclusion. They will discuss and resolve disagreements, if necessary involving a third reviewer (AC). The study selection process is depicted in Figure 1.

### Data Extraction

Reviewers will extract data from eligible articles with the use of a systematic review management software called Covidence (Veritas Health Innovation Ltd). The research team will develop a data extraction template to capture the items such as study information, study participants’ characteristics, characteristics of included primary studies, how the ASRH outcomes of interest are defined, factors associated with the outcomes of interest, and intervention models used (if relevant) and their influence on ASRH outcome. Factors under consideration include but are not limited to age, sex, marital status, schooling level, employment status, wealth, religion, knowledge about ASRH-related topics, age of sexual initiation, various behaviors such as the use of contraception or engagement in transactional sex, rural versus urban dwelling, parental care and communication, parental education, media influence, gender and cultural norms, and policies. For each included article, 2 reviewers (MZG and KO) will independently complete an extraction template, documenting all responses in Covidence. A third reviewer (AC) will resolve any disagreements about data extraction.

We will assess the methodological quality of the included articles using the Joanna Briggs Institute Critical Appraisal Checklist for Systematic Reviews and Research Syntheses, which covers both quantitative and qualitative research. Two reviewers will conduct the assessments independently and in pairs, and a third reviewer will resolve disagreements. The umbrella review will report the findings of the risk of bias assessment.

### Data Synthesis

The umbrella review will present a narrative synthesis of a range of ASRH outcomes and their associated factors. We will not attempt a meta-analysis of quantitative findings. We will organize the data by ASRH outcome—including pregnancy, HIV/AIDS transmission, intimate partner violence, and others—and synthesize the factors that have been explored in systematic reviews for each outcome. The review will also present subgroup analyses about associations of factors such as sex, age, school attendance, and rural versus urban dwelling with ASRH in sub-Saharan Africa.

### Ethical Considerations

This protocol follows the PRISMA-P (Preferred Reporting Items for Systematic Reviews and Meta-Analyses Protocols) guidelines [26]. The review is registered with PROSPERO (CRD42023394512). The review will exclusively use data from secondary sources and will not involve interactions with human subjects. Ethical clearance is not required. The findings will form part of the evidence to be discussed in a larger project to co-design strategies for improving ASRH in Bauchi State, Nigeria. The research team will also disseminate findings in conference presentations and publications.

## Results

The initial academic and gray literature search started in April 2023 and has resulted in a total of 1358 and 212 articles, respectively. After the removal of duplicates, 1351 articles remain to be screened. We expect to publish the review findings before the end of 2023.

## Discussion

### Summary

This is the first umbrella review within the sub-Saharan African context to assess comprehensively ASRH outcomes and the factors associated with them. By systematically reviewing both academic and grey literature, we aim to identify, synthesize, and critically evaluate existing research evidence on factors related to ASRH outcomes within the region. Existing systematic reviews focus on specific aspects of ASRH, but these are often intertwined so a broad overview across different outcomes is timely. By synthesizing the findings from multiple systematic reviews, this review will identify gaps in the existing evidence base. This can guide future research to fill these gaps.

The literature about ASRH in sub-Saharan Africa is diverse and complex, covering a range of outcomes, associated factors, and interventions. Many systematic reviews have synthesized the literature relating to different outcomes; our preliminary search strategy resulted in 1351 articles to be screened. An umbrella review is timely to synthesize existing systematic reviews and meta-analyses and will provide a comprehensive overview of the available evidence. Sub-Saharan Africa has limited human and financial resources. An umbrella review can guide the efficient allocation of scarce resources by identifying the most robust and relevant evidence, minimizing duplication of effort, and identifying areas where further research or interventions are needed.

Anticipated limitations of this review include the diversity and varying quality of the included reviews. We will consider the quality of the included reviews in our interpretation of the findings of our umbrella review. Another limitation is the potential overlap of original articles between the reviews included within our umbrella review. Ignoring potential overlap might lead umbrella reviews to overstate certain results and conclusions. We will identify overlaps in our synthesis report.

### Conclusions

This umbrella review of factors related to ASRH outcomes in sub-Saharan Africa is timely. It will provide a comprehensive overview of the landscape of studies on ASRH outcomes and potential determinants in sub-Saharan Africa. It will identify gaps in the evidence base and guide future research on the topic.

## Appendix

Multimedia Appendix 1 Peer review reports.

## Abbreviations

ASRH: adolescent sexual and reproductive health
PRISMA-P: Preferred Reporting Items for Systematic Reviews and Meta-Analyses Protocol
PROSPERO: International Prospective Register of Systematic Review
STD: sexually transmitted disease

## Data Availability

Data sharing is not applicable to this paper as no data sets were generated or analyzed during this study.
